# Supplementary material for: A Likelihood Approach for Real-Time Calibration of Stochastic Compartmental Epidemic Models
Source: PLoS Comput Biol. 2017 Jan 17;13(1):e1005257. doi: 10.1371/journal.pcbi.1005257 (PMC5240920; doi:10.1371/journal.pcbi.1005257)
Supplement: S1 File — (TAR.GZ) [file pcbi.1005257.s014.tar.gz › HSPH_Online-SI-Revision/Additions/EnKF08/n100-extreme-SEITR/n100-extreme-SEITR_EnKF_table4.pdf]

1	R0	{1.38654, 9.85565}	{2.26482, 4.68125}	{{1.32874, 3.22061}, {3., 5.87219}}	{66.9864, 52.5018}	inf	602.	7333.82	{3604.12, 7746.06}	67.8315	0.257794	newly	2747.	8729.44	{4633., 17097.}	224.941	0.483989	Reff	0.128602	0.572267	{0.206069, 1.15898}	349.068	ar	92379.	185246.	{163366., 237772.}	100.528
2	R0	{2.36629, 12.2029}	{2.53871, 5.93765}	{{1.86906, 0.525196}, {3., 16.4688}}	{19.4657, 68.7848}	inf	104.	9948.45	{1990.71, 23199.8}	99.015	0.268727	newly	2664.	14473.6	{1538., 26588.}	463.677	0.540315	Reff	0.0301229	0.554503	{0.156435, 1.11415}	1743.99	ar	99022.	204135.	{184452., 261141.}	106.151
3	R0	{2.32253, 12.836}	{2.13274, 11.3235}	{{0.58163, 3.74461}, {3., 19.328}}	{44.9254, 48.0223}	inf	185.	5988.35	{1001.86, 16308.3}	70.4945	0.416297	newly	3413.	3471.1	{411., 11359.}	75.305	0.365706	Reff	0.035767	0.317506	{0.0803029, 0.527891}	790.862	ar	98997.	181441.	{167476., 250336.}	83.279
4	R0	{1.79075, 11.2058}	{2.17809, 5.90394}	{{1.16826, 1.47452}, {3., 8.23181}}	{52.6501, 47.3135}	inf	424.	7661.7	{1752.32, 13993.4}	69.9393	0.300319	newly	3304.	8752.31	{1271., 18270.}	187.647	0.347019	Reff	0.0739401	0.517448	{0.133755, 0.878863}	608.125	ar	96920.	201465.	{182884., 231834.}	107.867
5	R0	{2.26952, 9.62371}	{1.7023, 9.15423}	{{0.245942, 3.7312}, {3., 19.1295}}	{57.9793, 49.0918}	inf	39.	3770.54	{1427.93, 4221.66}	77.462	0.171882	newly	1318.	2223.17	{545., 2798.}	93.1419	0.257773	Reff	0.0289591	0.28687	{0.0395652, 0.508167}	891.628	ar	98881.	184763.	{177181., 193962.}	86.8541
6	R0	{1.57191, 6.89004}	{2.33328, 3.94669}	{{1.82515, 3.05912}, {3., 4.34789}}	{55.7417, 45.4864}	inf	100.	6194.68	{1339.01, 16902.1}	335.688	0.426682	newly	1057.	9684.51	{1667., 22543.}	820.553	0.653619	Reff	0.0825567	0.666521	{0.245976, 1.82514}	715.374	ar	95046.	195426.	{171489., 257869.}	105.612
7	R0	{1.99226, 17.2339}	{2.34131, 4.46664}	{{1.31058, 2.85237}, {3., 6.23541}}	{44.1908, 74.0822}	inf	602.	7769.16	{6457.62, 7883.52}	43.204	0.242635	newly	3977.	11591.	{5211., 17549.}	195.326	0.370725	Reff	0.0686533	0.655714	{0.210669, 1.27862}	857.562	ar	97992.	181233.	{156787., 229860.}	84.9463
8	R0	{2.63369, 15.1879}	{2.38132, 6.58592}	{{1.70955, 0.588706}, {3., 17.0457}}	{23.9896, 63.4522}	inf	100.	18920.4	{3952.76, 51755.6}	155.824	0.392243	newly	3068.	23171.9	{2032., 87380.}	670.187	0.685342	Reff	0.022123	0.756801	{0.198893, 1.70976}	3322.98	ar	99451.	210939.	{171182., 293932.}	112.103
9	R0	{2.53271, 9.90552}	{1.83315, 9.92065}	{{0.200554, 3.73493}, {3., 19.5916}}	{48.9938, 52.6109}	inf	27.	6706.42	{2144.07, 14463.8}	171.408	0.206946	newly	1330.	4552.34	{802., 9800.}	263.892	0.339438	Reff	0.0191979	0.317358	{0.0331413, 0.547}	1553.09	ar	99355.	185137.	{172157., 229414.}	86.3384
10	R0	{2.48791, 18.9003}	{2.22601, 5.89488}	{{0.599017, 1.02455}, {3., 7.34141}}	{38.1083, 68.8107}	inf	387.	13706.2	{1083.2, 45426.}	93.7038	0.521432	newly	4707.	11692.3	{701., 37260.}	182.556	0.404246	Reff	0.0381148	0.454177	{0.0836796, 0.753549}	1092.36	ar	99291.	194070.	{163681., 271983.}	95.4554
11	R0	{2.84414, 18.4765}	{2.66417, 7.8101}	{{2.11809, 3.66011}, {3., 9.21646}}	{13.0734, 57.7296}	inf	161.	6136.91	{428.265, 13488.8}	83.8903	0.785127	newly	4255.	5434.77	{348., 9159.}	111.74	0.512313	Reff	0.0214164	0.80103	{0.409372, 2.11807}	3642.16	ar	99654.	178136.	{151554., 245169.}	78.7543
12	R0	{2.29369, 14.622}	{2.25602, 9.85173}	{{0.720829, 3.63519}, {3., 12.2958}}	{37.0871, 32.6239}	inf	189.	12482.2	{3606.96, 27214.3}	82.4624	0.232937	newly	3139.	10603.6	{2918., 26303.}	251.556	0.375768	Reff	0.0347723	0.571911	{0.0245845, 2.33853}	1559.29	ar	98982.	209533.	{182466., 278371.}	111.688
13	R0	{2.73413, 16.7529}	{2.3245, 6.11253}	{{1.16525, 3.45577}, {3., 7.54932}}	{26.2391, 63.5136}	inf	128.	11740.4	{2442.72, 16351.2}	74.1812	0.28196	newly	3594.	12397.6	{2542., 22918.}	259.001	0.400187	Reff	0.0212989	0.54328	{0.100953, 1.15998}	2453.52	ar	99543.	216640.	{191328., 267191.}	117.635
14	R0	{1.79554, 16.5943}	{2.28529, 3.31302}	{{0.831007, 2.93335}, {3., 4.17023}}	{55.904, 80.0352}	inf	1182.	11444.1	{1667.35, 29493.}	78.945	0.491965	newly	4850.	13432.4	{2006., 31871.}	207.697	0.43908	Reff	0.106063	0.435996	{0.151051, 0.797792}	313.406	ar	96821.	202160.	{167205., 284770.}	108.797
15	R0	{1.91429, 13.7771}	{2.2124, 5.35983}	{{1.20372, 3.44446}, {3., 6.21599}}	{47.9492, 61.0961}	inf	383.	4290.55	{3628.17, 6360.57}	50.9312	0.341956	newly	3380.	4938.78	{2658., 12457.}	67.9373	0.194036	Reff	0.0667896	0.504587	{0.103057, 1.21845}	659.534	ar	97541.	191373.	{175727., 216831.}	96.1977
16	R0	{1.42094, 10.5871}	{2.60064, 9.2879}	{{1.82061, 0.049993}, {3., 11.5721}}	{84.9064, 21.6246}	inf	1054.	4282.96	{703.909, 8806.18}	57.0526	0.436648	newly	4015.	3380.67	{488., 8083.}	69.4503	0.434642	Reff	0.134932	0.66862	{0.149653, 1.6788}	400.753	ar	93044.	176269.	{156892., 246512.}	89.4467
17	R0	{1.45515, 6.16466}	{2.07435, 12.4059}	{{0.294617, 6.76494}, {3., 19.1297}}	{96.4744, 102.039}	inf	170.	6312.85	{1080.31, 9920.54}	278.852	0.442644	newly	1376.	3686.71	{381., 6083.}	199.043	0.405488	Reff	0.105091	0.457231	{0.0423013, 0.639284}	366.667	ar	93254.	191060.	{173718., 249888.}	104.881
18	R0	{2.85669, 13.2416}	{2.32462, 5.8226}	{{1.86471, 4.21476}, {3., 12.7108}}	{23.1403, 56.028}	inf	31.	9870.62	{1000.42, 16934.6}	110.195	0.354162	newly	2583.	10658.7	{1047., 21390.}	337.227	0.511464	Reff	0.014512	0.607337	{0.134529, 1.76532}	4085.07	ar	99644.	200459.	{173222., 254333.}	101.175
19	R0	{2.56417, 17.9558}	{2.52219, 11.3411}	{{1.66951, 3.43433}, {3., 19.0481}}	{23.3933, 40.1345}	inf	266.	4909.21	{525.132, 9458.45}	79.9918	0.799573	newly	4524.	3476.56	{250., 9719.}	84.8046	0.614283	Reff	0.0335137	0.59779	{0.16796, 1.2917}	1684.09	ar	99389.	169337.	{151680., 193301.}	70.3783
20	R0	{2.27517, 11.1864}	{2.46661, 6.98196}	{{1.82179, 4.26069}, {3., 11.4833}}	{26.3489, 38.9123}	inf	92.	8661.97	{958.933, 14896.2}	143.001	0.396662	newly	2013.	8445.13	{889., 14695.}	344.838	0.495631	Reff	0.0313746	0.54149	{0.0814274, 1.37536}	1628.66	ar	98894.	192705.	{169526., 243683.}	94.8604
21	R0	{2.35735, 10.4735}	{1.75897, 10.4003}	{{0.185967, 3.74124}, {3., 19.0685}}	{54.8263, 52.0137}	inf	65.	4445.53	{1189.19, 7730.14}	66.0444	0.242138	newly	1848.	2730.17	{643., 7387.}	86.8728	0.290159	Reff	0.0275103	0.255983	{0.0308342, 0.492207}	831.211	ar	99036.	176675.	{166926., 186961.}	78.3944
22	R0	{1.79551, 11.9179}	{2.17581, 3.59627}	{{0.508621, 2.8782}, {3., 4.91692}}	{68.7111, 69.8247}	inf	355.	10649.2	{1372.33, 24563.4}	112.044	0.32143	newly	2882.	10708.3	{1477., 25426.}	291.507	0.487199	Reff	0.0710663	0.29886	{0.0971215, 0.524218}	323.666	ar	97033.	188967.	{164779., 244999.}	94.7454
23	R0	{1.23545, 6.31687}	{2.59113, 11.2029}	{{1.82474, 4.88554}, {3., 15.2228}}	{109.731, 91.8222}	inf	264.	11392.4	{538.726, 25056.5}	608.261	0.668844	newly	1370.	7887.03	{295., 21668.}	501.196	0.569249	Reff	0.148353	0.648218	{0.209941, 1.81309}	338.466	ar	88739.	205359.	{177153., 280068.}	131.419
24	R0	{2.24089, 10.1789}	{2.36489, 7.37552}	{{1.80963, 4.27369}, {3., 11.9447}}	{25.5481, 38.2965}	inf	88.	8604.51	{1826.89, 18569.9}	133.198	0.329129	newly	2182.	8230.13	{1498., 19408.}	291.182	0.427724	Reff	0.0325825	0.57498	{0.154936, 1.68759}	1664.69	ar	98868.	200066.	{177272., 252232.}	102.356
25	R0	{1.57298, 10.2607}	{2.49196, 7.29834}	{{0.610452, 3.6111}, {3., 8.32089}}	{76.3893, 28.8709}	inf	633.	3796.81	{333.414, 14066.2}	85.2361	0.706238	newly	3489.	3819.02	{245., 11663.}	116.671	0.641432	Reff	0.10572	0.559515	{0.120117, 0.679801}	437.707	ar	94957.	168922.	{152544., 199753.}	77.8928
26	R0	{1.2154, 8.2576}	{2.45709, 10.8587}	{{0.956149, 4.98898}, {3., 13.4701}}	{111.83, 52.0401}	inf	684.	8169.06	{891.053, 15717.4}	152.127	0.394987	newly	2648.	4973.45	{444., 9465.}	142.789	0.416954	Reff	0.165416	0.624216	{0.16647, 0.958236}	285.702	ar	88421.	180008.	{158745., 223745.}	103.58
27	R0	{2.22841, 18.7983}	{2.50695, 6.77204}	{{0.881401, 3.4023}, {3., 9.92346}}	{40.1485, 63.9753}	inf	454.	5552.23	{2269.78, 9446.62}	69.2274	0.518293	newly	4125.	6716.8	{1466., 11112.}	129.143	0.387555	Reff	0.0518997	0.498191	{0.179733, 0.62945}	859.912	ar	98756.	179921.	{167122., 197761.}	82.1872
28	R0	{1.27778, 5.36767}	{2.08343, 9.51401}	{{0.217077, 3.73563}, {3., 19.5303}}	{117.272, 91.2328}	inf	160.	5069.44	{1768.37, 7655.63}	358.967	0.479159	newly	965.	3972.02	{864., 4819.}	324.276	0.417779	Reff	0.136275	0.332431	{0.0341421, 0.574505}	182.422	ar	89843.	182045.	{173332., 200792.}	102.625
29	R0	{2.38042, 15.8901}	{2.34819, 3.5536}	{{1.04188, 3.14199}, {3., 4.65056}}	{32.5879, 77.6364}	inf	266.	9206.53	{1374.83, 15998.7}	68.1907	0.450676	newly	4278.	12473.1	{1679., 32786.}	219.9	0.43498	Reff	0.0399673	0.578725	{0.278884, 0.893894}	1348.	ar	99112.	192327.	{171061., 236589.}	94.0497
30	R0	{2.53188, 12.7607}	{1.86868, 10.3606}	{{0.52283, 3.03125}, {3., 19.6309}}	{45.7925, 53.9419}	inf	140.	4103.91	{628.079, 4858.27}	63.9252	0.507479	newly	3519.	2793.52	{462., 5404.}	63.3492	0.431916	Reff	0.0262556	0.288405	{0.0545578, 0.60884}	1000.86	ar	99317.	178439.	{171458., 181478.}	79.666
31	R0	{1.24482, 10.5471}	{2.25698, 4.48735}	{{1.34574, 3.5882}, {3., 4.27408}}	{81.31, 60.4008}	inf	1178.	19448.	{3165.35, 58601.5}	248.497	0.442306	newly	3288.	22169.9	{3231., 60845.}	584.345	0.673531	Reff	0.174511	0.625972	{0.231929, 1.10009}	259.502	ar	89097.	225560.	{179225., 305443.}	153.163
32	R0	{2.30446, 17.9607}	{2.1131, 7.15436}	{{0.25035, 5.52922}, {3., 7.84298}}	{46.9374, 60.1666}	inf	386.	4026.71	{602.878, 7326.9}	76.7404	0.767781	newly	4308.	3050.65	{506., 6529.}	70.3489	0.479838	Reff	0.0453748	0.386609	{0.0867832, 0.707085}	753.361	ar	99028.	176632.	{167703., 196977.}	78.3654
33	R0	{2.31579, 11.8236}	{1.95026, 8.06}	{{0.255008, 3.71398}, {3., 19.0747}}	{51.8296, 51.9039}	inf	79.	3112.45	{856.421, 2976.89}	66.1394	0.364994	newly	2012.	2462.45	{669., 3301.}	72.9587	0.248955	Reff	0.0280905	0.342363	{0.0421694, 0.503958}	1118.78	ar	99012.	173976.	{166842., 173711.}	75.712
34	R0	{1.6678, 10.6744}	{2.29479, 4.09048}	{{0.708771, 2.72122}, {3., 7.50788}}	{68.2364, 61.6795}	inf	325.	9682.74	{1975.18, 20075.5}	139.098	0.276054	newly	2446.	10477.6	{2047., 30211.}	336.793	0.464779	Reff	0.0850078	0.513966	{0.146015, 0.716319}	506.831	ar	95809.	191675.	{165793., 256546.}	100.059
35	R0	{1.65476, 7.07559}	{1.97751, 12.2898}	{{0.180151, 7.01843}, {3., 19.5275}}	{83.0158, 81.9341}	inf	106.	4018.56	{1988.11, 4336.85}	146.47	0.33639	newly	1151.	2133.16	{668., 3285.}	109.336	0.318965	Reff	0.0754074	0.308539	{0.0255108, 0.473555}	353.141	ar	95746.	175778.	{165036., 214191.}	83.5879
36	R0	{2.71807, 18.5959}	{2.06143, 6.77781}	{{0.64312, 5.38023}, {3., 7.47622}}	{36.6053, 63.5521}	inf	279.	5918.85	{453.172, 12321.2}	80.7069	0.816913	newly	5029.	5038.54	{299., 11956.}	100.05	0.567022	Reff	0.0291921	0.453611	{0.0946537, 0.788554}	1453.89	ar	99540.	187220.	{169168., 215459.}	88.0853
37	R0	{2.6202, 12.7552}	{2.31165, 3.66959}	{{1.40423, 3.22877}, {3., 4.6064}}	{26.5032, 71.2306}	inf	74.	16814.2	{1361.86, 39915.9}	187.192	0.486301	newly	2840.	26437.	{1321., 78022.}	847.073	0.801536	Reff	0.0220097	0.753094	{0.291815, 1.58365}	3321.65	ar	99426.	232579.	{191252., 310516.}	133.921
38	R0	{1.47672, 9.81651}	{2.572, 9.28813}	{{1.79343, 7.43673}, {3., 10.4838}}	{74.1699, 11.179}	inf	503.	5655.57	{354.397, 16602.7}	116.284	0.531514	newly	2643.	4101.98	{194., 14042.}	134.807	0.493291	Reff	0.109425	0.714307	{0.308108, 1.77382}	553.876	ar	93815.	173423.	{151349., 255607.}	84.8564
39	R0	{1.90004, 12.3313}	{1.88503, 6.99451}	{{0.242202, 5.46024}, {3., 7.7505}}	{65.6284, 43.2784}	inf	369.	4659.05	{624.558, 12075.5}	77.0814	0.524167	newly	3317.	3661.18	{411., 8773.}	89.6629	0.400764	Reff	0.0681354	0.396993	{0.0327313, 0.747246}	500.235	ar	97413.	178801.	{164772., 203888.}	83.5493
40	R0	{2.64218, 18.5237}	{2.38315, 6.04149}	{{1.42645, 3.40173}, {3., 7.15968}}	{24.9862, 67.3851}	inf	174.	11027.7	{966.657, 9366.77}	88.9538	0.431261	newly	3674.	9419.6	{1183., 8940.}	176.04	0.323244	Reff	0.0267917	0.462395	{0.112898, 0.858269}	1629.67	ar	99474.	201142.	{178803., 246516.}	102.205
41	R0	{1.6945, 7.17996}	{1.87382, 12.1216}	{{0.414109, 6.73856}, {3., 18.1714}}	{74.1652, 74.8504}	inf	141.	5619.79	{1093.36, 8679.43}	147.723	0.31766	newly	1635.	3250.94	{377., 3411.}	139.354	0.364307	Reff	0.0694067	0.320662	{0.0736084, 0.565426}	362.836	ar	96332.	190193.	{179169., 221280.}	97.4352
42	R0	{1.84559, 18.0074}	{2.31276, 4.35368}	{{1.1371, 1.89868}, {3., 7.78267}}	{53.5, 75.8228}	inf	1048.	10281.	{814.451, 30543.8}	88.0474	0.575531	newly	4650.	15221.2	{1249., 55430.}	257.004	0.43643	Reff	0.093313	0.785578	{0.304796, 1.19556}	745.717	ar	97358.	197594.	{166548., 273736.}	102.956
43	R0	{2.24965, 17.0361}	{2.00248, 7.27087}	{{0.607405, 5.53914}, {3., 7.86946}}	{49.0108, 57.3208}	inf	520.	5009.97	{671.375, 14789.8}	73.178	0.716838	newly	4598.	3809.41	{537., 9612.}	82.1512	0.499722	Reff	0.0537891	0.452334	{0.0260502, 0.687307}	759.975	ar	98770.	190779.	{174534., 220526.}	93.1552
44	R0	{2.54324, 15.7967}	{2.13617, 3.80369}	{{0.595176, 2.95022}, {3., 6.03988}}	{38.9946, 75.921}	inf	127.	12895.	{2154.56, 39826.5}	102.189	0.341296	newly	3246.	12768.8	{2457., 28647.}	303.717	0.451684	Reff	0.0255087	0.318884	{0.0934918, 0.54061}	1157.02	ar	99354.	202233.	{172379., 265624.}	103.548
45	R0	{2.54943, 14.8032}	{2.44603, 6.7649}	{{1.86806, 0.474753}, {3., 17.63}}	{19.9619, 64.6019}	inf	120.	6452.55	{448.67, 6721.1}	68.9546	0.445543	newly	3187.	14195.9	{1368., 40687.}	385.521	0.563609	Reff	0.0250609	0.710584	{0.219204, 1.77199}	2736.71	ar	99380.	204361.	{180071., 267601.}	105.636
46	R0	{2.37755, 12.6461}	{1.58407, 8.24618}	{{0.19093, 3.72928}, {3., 19.774}}	{58.507, 54.6329}	inf	129.	5359.07	{922.703, 8550.66}	79.7478	0.465165	newly	3124.	4507.85	{553., 7180.}	115.717	0.369131	Reff	0.0309795	0.213118	{0.00498611, 0.65962}	622.727	ar	99096.	181809.	{170553., 204827.}	83.4671
47	R0	{1.69279, 11.6636}	{2.55098, 7.25524}	{{2.12866, 3.59609}, {3., 8.62386}}	{62.0522, 37.7959}	inf	535.	2627.53	{251.582, 4020.8}	85.2207	0.830109	newly	3657.	2210.15	{206., 4719.}	86.1031	0.634962	Reff	0.0899041	0.657269	{0.0896298, 1.06766}	639.709	ar	96214.	172519.	{159670., 192191.}	79.3071
48	R0	{1.82402, 10.0038}	{2.62408, 6.53082}	{{1.99781, 0.502656}, {3., 17.5728}}	{43.8623, 74.0569}	inf	312.	10963.5	{1402.2, 30563.1}	149.219	0.342654	newly	2763.	15414.4	{1577., 46396.}	476.918	0.492324	Reff	0.0676164	0.628533	{0.263851, 1.44069}	832.386	ar	97076.	219840.	{190473., 275847.}	126.462
49	R0	{1.6424, 11.1667}	{2.2147, 4.31408}	{{0.69509, 2.52645}, {3., 8.60712}}	{71.6901, 61.3666}	inf	368.	12232.9	{2581.43, 27589.8}	173.51	0.308374	newly	2454.	13922.2	{2606., 38363.}	475.846	0.571259	Reff	0.0891166	0.532168	{0.0818964, 1.32826}	502.927	ar	95546.	195228.	{165508., 283359.}	104.329
50	R0	{2.05638, 14.3048}	{2.48515, 6.16524}	{{1.31684, 3.36701}, {3., 7.25543}}	{40.0133, 56.9009}	inf	362.	9964.42	{758.776, 11422.3}	84.913	0.37973	newly	3752.	8223.56	{1006., 11274.}	141.367	0.335583	Reff	0.0564271	0.498778	{0.226478, 0.826477}	784.112	ar	98194.	190276.	{169478., 215534.}	93.7761
